# Interplay between cancer-associated fibroblasts and dendritic cells: implications for tumor immunity

**DOI:** 10.3389/fimmu.2025.1515390

**Published:** 2025-05-16

**Authors:** Fátima María Mentucci, María Gracia Ferrara, Agustina Ercole, Natalia Belén Rumie Vittar, María Julia Lamberti

**Affiliations:** ^1^ Instituto de Biotecnología Ambiental y Salud (INBIAS UNRC CONICET)-UNRC, Río Cuarto, Córdoba, Argentina; ^2^ Departamento de Biología Molecular, Facultad de Ciencias Exactas, Físico-Químicas y Naturales, Universidad Nacional de Río Cuarto, Río Cuarto, Córdoba, Argentina

**Keywords:** tumor microenvironment, cancer, stroma, immunotherapy, oncoimmunology, immunosuppression, tolerogenicity

## Abstract

The tumor microenvironment (TME) plays a critical role in cancer progression, with cancer-associated fibroblasts (CAFs) emerging as key players in immune evasion. This review explores the complex interactions between CAFs and dendritic cells (DCs), essential antigen-presenting cells that activate immune responses. CAFs impair DC maturation and function by secreting cytokines, chemokines, and growth factors, reducing their ability to present antigens and stimulate T cells, thus promoting an immunosuppressive environment favorable to tumor growth. Additionally, CAFs contribute to the differentiation of tolerogenic DCs, fostering regulatory T cells (Tregs) that further suppress antitumor immunity. This review examines the molecular mechanisms underlying CAF-DC crosstalk and discusses potential therapeutic strategies aimed at restoring DC functionality. Targeting the CAF-driven immunosuppressive network offers promising opportunities to enhance the efficacy of DC-based vaccines and immunotherapies, paving the way for improved cancer treatment outcomes.

## 1 Introduction: tumor microenvironment as a dynamic ecosystem driving cancer progression

Cancer has been described as one of the most complex diseases known. This complexity stems from several factors contributing to the intricate nature for development. The persistent statistic positioning cancer as the cause of death demonstrates that, despite the vast body of research revealing the multifaceted mechanisms of carcinogenesis, this underlying complexity remains only partially understood (1, 2). The shift in research perspective from reductionist biology to a multidimensional understanding, with the recognition of the hallmarks of cancer, demonstrates the need for cooperative actions by tumor cells to proliferate, expand, and disseminate. Additionally, for over a century, the inevitable accumulation of mutations has been recognized as the driving force behind the formation of tumor cells, establishing cancer as a genetic disease. However, this explanation is insufficient, as evidence shows that a cell with oncogenic mutations requires a specific context to initiate a tumor. Technological advancements in biological research reveal that tumor cells reside in a dynamic environment called the tumor microenvironment (TME), which reflects their ecosystem. Considering the complexity of the disease, it becomes evident that it arises from the TME. Within this environment, the tumor cells cohabitate with various non-tumoral populations embedded all in an extracellular matrix to ultimately form the tumor mass. This non-cancerous counterpart is typically composed of diverse resident or recruited adaptive and innate immune cell types, cancer-associated fibroblasts (CAFs), blood vessels such as endothelial cells and pericytes, and various additional tissue-specific cell types (such as neurons, adipocytes, melanocytes, keratinocytes, among others, depending on the organ of origin), which also contributed to tumor coevolve (1–3). The process of dynamic evolution is based on the dialogues established between the intervening populations through signaling at two levels: indirect paracrine signaling or direct cell-to-cell contact (3). These interactions, which are established in a spatially and temporally regulated manner, create niches within the microenvironment that govern its functional state. The functionality of the TME continuously transitions to support the intrinsic properties of the tumor cells residing within, resulting in distinct states that drive tumor growth, invasion, and metastasis. Thus, the composition and functionality of a dynamic TME are shaped by the organ of origin, tumor stage, and the patient’s characteristics (3). This habitat creates a tumor-suppressive or supportive environment, which correlates with treatment response and immune surveillance. Our understanding of the TME, with focusing on stromal composition, represents a challenge for exploring vulnerabilities and proposing them as therapeutic strategies.

As previously mentioned, the TME is a highly specialized ecosystem that arises, shapes, and evolves is driven by tumor cells which one acquired capabilities to recruit and corrupt both the normal cellular and non-cellular components of their emerging surroundings. The nature of the tumor dictates the specific stromal drivers involved in its progression and the dynamic and heterogeneous plays a critical role in encompassing all stages of tumor development (3–5). An enhanced tumor-supportive TME serves as a dynamic reservoir of cellular and extracellular components, intricately involved in cancer outcome. Collectively, these elements are the tumor’s fuel for survival, invasiveness, and response to therapies, making the TME a critical determinant for prognosis and therapeutic intervention. For a comprehensive overview of the major cellular and non-cellular components of the TME, refer to de Visser and Joyce (3). Their work provides an in-depth analysis of the key contributors, including immune cells such as adaptive immune cells, myeloid immune cells, immune cells at the interface of adaptive and innate immunity, stromal cells and matrix and vascular cells. Additionally, therapeutic strategies targeting the stromal components of the TME are thoroughly reviewed offering insights into novel approaches to disrupt tumor-stroma interactions and enhance treatment efficacy (4, 6)

Regarding stromal TME counterparts, two populations stand out due to the extensive diversity of their subtypes and subpopulations: immune cells and transformed fibroblasts. These populations exhibit significant functional and phenotypic heterogeneity, highlighting their key roles in the regulation and remodeling required to the successful tumor demands. In the following paragraphs, these aspects will be explored in depth.

Next year marks the 20th anniversary of the first scientific evidence establishing CAFs as critical contributors to cancer progression, acting as active components of the TME (7). Since that landmark discovery, numerous studies have consistently reinforced and expanded this evidence, underscoring the pivotal role of CAFs in tumor biology and highlighting their potential as therapeutic targets (4, 5, 8–12).

While the existence of CAFs is widely recognized, it actually remains a subject of debate about their precise role within TME. Over the past few decades, numerous studies have reported both pro-tumorigenic and anti-tumorigenic functions of CAFs, with attempts at therapeutic targeting yielding conflicting results. These endeavors often failed to account for the extensive heterogeneity and plasticity inherent in the CAF population, a complexity that is only now being fully appreciated. This variability may reflect diverse origins and specialized functions acquired by CAFs in response to specific environment cues and the tumor modeling received within the TME (4, 12).

Given the described role of this population, it is unsurprising that it predominates in the tumor niche, with some exceptions where its presence is null or minimal, such as in hematological cancers or glioblastomas, respectively, due to the natural lack of fibroblasts in these tissues (5, 11–14).

On the other side, the immune cells are abundantly present in the tumor with the intention to defend against the body to the malignant tissue. However, this response is unsuccessful due to the inevitable interstromal interactions with CAFs, which result dominate and create an immunosuppressive condition. The intricate interactions between stromal cells CAFs and various immune cells are thoroughly revised in detail by Xu et al. (4). Among the actions performed by them, we can mention remodeling the ECM to create a physical barrier for immune cell transit, modulating the antitumor activity of immune infiltrates, and enhancing the expression of checkpoint molecules (4, 11, 12). This condition is strongly maintained by tumor cells, which result in evading immune system attacks.

The objective of this review is to investigate the intricate and often enigmatic interactions within the TME, particularly focusing on the roles of immune cells and CAFs. By delving into the complexities of tumor immune surveillance and the potential barriers to effective antitumor responses, this review seeks to uncover the underlying mechanisms that could reveal new therapeutic avenues for cancer treatment.

## 2 Crucial role and function of immune cells in the TME

Tumor immune surveillance is a critical mechanism through which the immune system identifies and eliminates transformed cells before they can develop into clinically significant tumors. This process involves various immune cells from both the innate and adaptive immune systems, each playing distinct roles in recognizing and responding to tumor cells (15). The effectiveness of tumor immune surveillance can be understood through the three E’s theory: Elimination, Equilibrium, and Escape. In the elimination phase, the immune system effectively recognizes and destroys nascent tumor cells, preventing their growth. The equilibrium phase follows, where surviving tumor cells may enter a dormant state, controlled by the immune system. Lastly, in the escape phase, this equilibrium can be disrupted, allowing tumor cells to develop mechanisms to evade immune detection and suppress immune responses, leading to tumor growth and progression (16–18).

The innate immune system provides the first line of defense against tumors and includes various cell types found within the TME. Natural Killer (NK) cells are crucial for recognizing and killing tumor cells that may have downregulated major histocompatibility complex (MHC-I) molecules, and they can also secrete cytokines that enhance the immune response. Additionally, Myeloid-Derived Suppressor Cells (MDSCs) expand in cancer and exert immunosuppressive effects, inhibiting T cell activation and promoting tumor growth, representing a significant barrier to effective immune surveillance (16–18). Tumor-associated neutrophils (TANs) also play important roles in tumor immune surveillance and progression. They can exhibit either tumor-suppressive (N1) or tumor-promoting (N2) phenotypes depending on signals in the TME, contributing to angiogenesis, extracellular matrix remodeling, and immunosuppression (19). γδ T cells are unconventional lymphocytes with potent cytotoxic functions and rapid cytokine release, capable of responding to stress-induced molecules on tumor cells (20). However, they may also acquire regulatory phenotypes that favor tumor growth. Monocytes, recruited from the bloodstream, can differentiate into tumor-associated macrophages or dendritic cells and modulate the immune milieu through the secretion of inflammatory or suppressive cytokines, thus playing dual roles in tumor immunity depending on their polarization and context. Macrophages, which can adopt different functional states such as pro-inflammatory (M1) or anti-inflammatory (M2), play a versatile role in the TME. Often, macrophages exhibit an M2-like phenotype that promotes tumor progression and suppresses effective immune responses (21).

The adaptive immune system, which provides long-lasting and memory immunity, is represented by T lymphocytes and B lymphocytes. CD8+ cytotoxic T cells are key players in directly killing tumor cells, while CD4+ helper T cells orchestrate immune responses by activating other immune cells. B lymphocytes contribute to the immune response by producing antibodies against tumor antigens and can participate in forming tumor-infiltrating lymphoid structures within the TME (16–18).

Regulatory T cells (Tregs) are a subset of CD4+ T cells characterized by their expression of the transcription factor FoxP3, playing a critical role in maintaining immune tolerance and preventing excessive immune responses. In the context of cancer, Tregs can significantly influence the immune landscape within the TME. They are often expanded and activated, contributing to immune suppression that inhibits the activity of effector T cells, such as CD8+ cytotoxic T cells, thereby promoting tumor growth. Tregs achieve this through several mechanisms, including the secretion of immunosuppressive cytokines like IL-10 and TGF-β, which dampen immune responses and promote an anti-inflammatory environment favorable to tumor survival. Additionally, Tregs can inhibit the activation and proliferation of other immune cells through direct contact and promote the differentiation of MDSCs and other immunosuppressive cell types, exacerbating the immunosuppressive TME (16–18).

Dendritic cells (DCs) serve as crucial mediators between the innate and adaptive immune systems within the TME. They capture tumor antigens from the microenvironment, process them, and present them to naïve T cells in the lymph nodes. By doing so, DCs bridge the initial innate immune response and the subsequent adaptive response, guiding the differentiation of T cells into effector cells capable of targeting tumor cells effectively (22–24)

Factors influencing tumor immune surveillance include the immunogenicity of the tumor, the presence of immune checkpoints, and the TME itself. A well-functioning immune response can lead to the recognition and destruction of tumors; however, tumors can develop various mechanisms to evade immune detection, such as downregulating antigen presentation, secreting immunosuppressive factors, and creating a hostile TME that inhibits immune cell function (16–18).

## 3 Function and significance of dendritic cells in the immune response: challenges faced by DCs in cancer

Dendritic cells (DCs) are pivotal in bridging the gap between the innate and adaptive immune systems, playing a vital role in shaping the quality and magnitude of the immune response. As the most potent antigen-presenting cells (APCs), DCs are essential for initiating and regulating T cell responses, which are critical for effective tumor immune surveillance (25).

Traditionally, the classification of DCs subsets has relied on their ontogeny; however, recent single-cell analyses are uncovering a broader spectrum of functional states of DCs in the context of cancer. Within the TME, several subsets of DCs are present, each with distinct functions that contribute to the immune landscape (26). Conventional dendritic cells (cDCs) are primarily responsible for capturing and presenting tumor antigens to T cells, facilitating their activation. These cells can be further divided into two subsets: cDC1 cells, which are primarily involved in cross-presentation of antigens to activate CD8+ T cells (27), and cDC2 cells, which are more effective at activating CD4+ T helper cells and producing cytokines that shape the immune response (28). In addition to cDCs, plasmacytoid dendritic cells (pDCs) are known for their ability to produce large amounts of type I interferons in response to viral infections and tumor-derived signals. In the TME, pDCs can exhibit both immunostimulatory and immunosuppressive functions, influencing the overall immune landscape and potentially contributing to tumor progression or suppression, depending on the context (29). Furthermore, monocyte-derived dendritic cells (mo-DCs) arise from circulating monocytes that differentiate in response to inflammatory signals present in the TME. They can play a dual role by acting as APCs while also contributing to immunosuppression, depending on the tumor context (30, 31). While both humans and mice possess conventional DCs (cDC1 and cDC2) and plasmacytoid DCs (pDCs), their phenotypic markers and developmental pathways show important divergences. For example, human cDC1 express CLEC9A, XCR1, and CD141, whereas murine cDC1 are typically defined by CD8α or CD103 expression, although both rely on BATF3 and IRF8 for development and are key players in cross-presentation and Th1 polarization. Similarly, human cDC2 express CD1c and SIRPα and depend on IRF4, while their murine counterparts are CD11b+ and also express SIRPα (32). Importantly, a novel subset termed DC3 has been described in humans, characterized by the co-expression of CD1c and CD14 and a distinct proinflammatory profile; however, no direct murine equivalent has been identified to date. DC3 arise from a low-IRF8 progenitor and are capable of inducing Th17 or Th1 responses depending on the context (33, 34).

Beyond their antigen-presenting capabilities, DCs produce various cytokines that influence T cell differentiation and activation. Depending on the signals they receive from their environment, DCs can polarize T cells toward different subsets, such as T helper (Th) 1 cells that promote a strong immune response or regulatory T cells (Tregs) that can suppress immune activity. Their ability to migrate to lymph nodes and stimulate T cells underscores their indispensable role in orchestrating effective immune responses against tumors and infections (35, 36).

Overall, the functional versatility of DCs in the TME highlights their significance in modulating immune responses. Understanding the diverse roles of DC subsets and their interactions within the TME is crucial for developing strategies to enhance tumor immunity and improve therapeutic outcomes. Recently, the role of a newly classified group of dendritic cells known as regulatory dendritic cells (regDC) has garnered significant attention in tumor immunology, highlighting their critical function in modulating the immune response against cancer. Emerging findings indicate that regDC can promote immune tolerance by suppressing effector T cells and facilitating the expansion of Tregs within the TME. This immunosuppressive activity enables tumors to evade immune detection and grow unchecked, presenting challenges for effective cancer treatment (37).

In the context of tumor immune surveillance, DCs play a crucial role in detecting and presenting tumor-associated antigens, initiating an effective antitumor immune response (38). However, in cancer patients, the functionality of DCs is often compromised due to the immunosuppressive nature of the TME. Factors such as cytokines, metabolites, and cell-to-cell interactions within the TME can lead to the maturation of dysfunctional DCs that fail to activate T cells effectively. This dysfunction may manifest in several ways: decreased expression of co-stimulatory molecules, impaired cytokine production, and reduced capacity for antigen presentation. Consequently, the immune system may not mount an adequate response against the tumor, allowing for tumor growth and progression (39).

The TME can indeed be a significant contributor to the challenges faced by DCs in cancer patients, as the presence of cancer-associated fibroblasts (CAFs), regulatory immune cells, and various soluble factors can create a hostile environment that impairs DC maturation and function. CAFs, in particular, play a pivotal role in shaping TME and can profoundly impact the behavior of DCs. Their interactions with DCs and other immune cells can lead to further suppression of the immune response, contributing to the overall immune evasion by tumors (40). Understanding these mechanisms is essential for developing strategies to enhance DC functionality and improve tumor immune surveillance. This knowledge will pave the way for novel immunotherapeutic approaches aimed at countering the adverse effects of the TME, particularly the influence of CAFs, which will be explored in the following section.

## 4 Cancer-associated fibroblasts and their role in the TME

Fibroblasts are critical players in tissue homeostasis, wound healing, fibrotic conditions, and cancer progression (41). In cancer, fibroblasts transition into CAFs, which become prominent components of the TME. CAFs can originate from diverse sources, including resident fibroblasts, mesenchymal stem cells (MSCs), epithelial cells, endothelial cells, pericytes, and adipocytes (9, 42). This plasticity allows fibroblasts to adapt to the TME, driven by signals such as TGFβ, PDGF, and FGF, which convert normal fibroblasts into CAFs (41).

**Figure 1:**
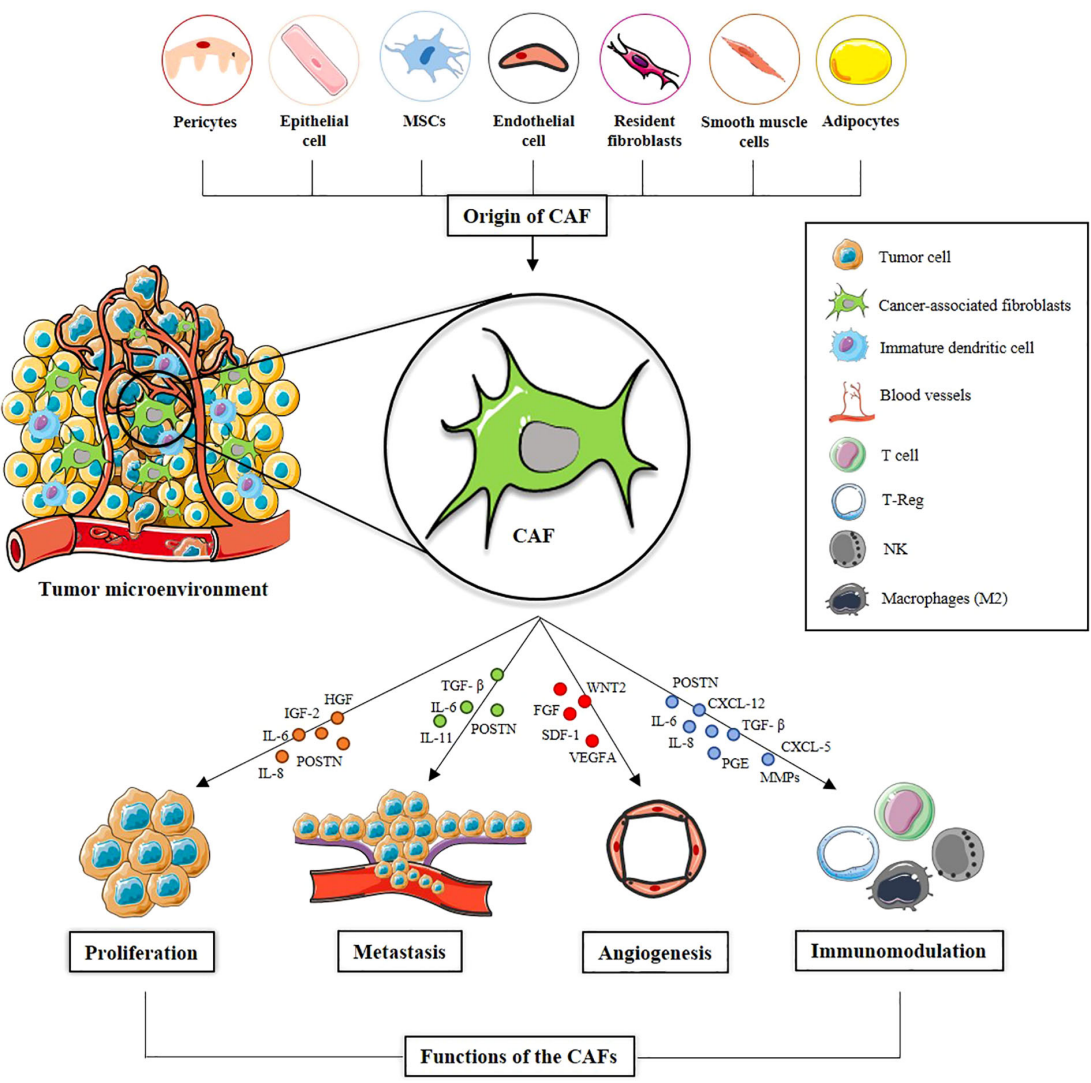
Origin and functions of cancer-associated fibroblasts (CAFs). CAFs represent a heterogeneous cell population within the TME and can originate from various cellular sources. Within TME, CAFs acquire an activated phenotype and secrete a wide range of soluble factors, including cytokines, growth factors, and extracellular matrix components. These mediators regulate key processes in tumor progression, including cancer cell proliferation, metastasis, angiogenesis, and immunomodulation, thereby fostering a tumor-promoting environment. The figure illustrates the main signaling pathways associated with each of these functions, highlighting the interactions between CAFs and other TME components.

The heterogeneity of CAFs manifests at multiple levels: cellular (function and activation state), microenvironmental (interactions with tumor cells and the extracellular matrix), and regional (tissue-specific functions), influencing tumor biology and treatment responses. Understanding this heterogeneity is crucial for the development of targeted therapies (9). CAFs play key roles in the TME by secreting a wide range of growth factors, inflammatory mediators, and extracellular matrix (ECM) components, which not only promote tumor growth but also contribute to therapy resistance and immune evasion (10) Through signaling interactions with cancer cells, CAFs drive processes such as proliferation, metastasis, angiogenesis, and immunomodulation, among others (42)(**Figure 1**).

Recent advances in single-cell RNA sequencing (scRNA-seq) and transcriptomics have revealed a high degree of heterogeneity in CAFs across different cancer types, enabling the identification of distinct CAF subpopulations that correlate with specific tumor profiles. This discovery enhances our ability to predict treatment responses and tailor personalized therapies (9). Although CAF nomenclature varies across studies and cancer types, these subpopulations can be grouped into well-defined functional and molecular categories that reflect their role in the TME. Over the years, studies in various cancer types have consistently identified at least two main CAF subtypes: myofibroblastic CAFs (myCAFs) and inflammatory CAFs (iCAFs), with multiple classification systems converging on these categories (43). myCAFs are characterized by high αSMA expression, which confers a contractile phenotype and a crucial role in extracellular matrix (ECM) remodeling. Although these CAFs have been identified as myCAFs in pancreatic ductal adenocarcinoma (PDAC) and lung cancer (44, 45), in other tumor types, they have been assigned different nomenclatures despite sharing similar functions and molecular signatures. For example, in melanoma, myCAFs have been designated as CAF-S2 (46), in colorectal cancer as CAF-B (47) and in breast cancer as mCAFs (48). Conversely, iCAFs exhibit a secretory profile characterized by the production of cytokines and growth factors, enabling them to modulate inflammation and tumor progression. They have been described in various tumors, including PDAC, melanoma, and breast cancer (46, 48, 49). These CAFs play a key role in shaping the immune landscape of the TME, as they induce chronic inflammation and contribute to an immunosuppressive environment that promotes tumor survival. Their secretion of cytokines such as TGF-β, IL-6, CXCL1, and CXCL12 suppresses the activity of cytotoxic CD8+ T cells and promotes the recruitment of immunosuppressive cells such as MDSCs (41).

Recently, a CAF subtype with immunomodulatory properties, antigen-presenting CAFs (apCAFs), has gained attention due to their presence in various solid tumors. Characterized by MHC-II expression, these fibroblasts suggest a crucial role in interacting with immune cells. To date, they have been described in pancreatic, lung, and breast cancer (45, 50–53). In PDAC, apCAFs have been shown to modulate immune responses, whereas in melanoma, they have been designated as CAF-S1 and perform a similar function in suppressing antitumor immune responses (46, 49). These findings highlight the remarkable diversity of CAFs, whose genetic and proteomic profiles vary among different tumor types, playing key roles in both cancer progression and therapeutic responses (9).

## 5 Mechanisms of CAF-mediated modulation of DCs

Given the critical role of DCs in innate immunity, their dysfunction in the TME, and the influence of CAFs in driving immunosuppression, we chose to focus this review on the interaction among these three key components. While DCs are essential for initiating anti-tumor immune responses (54, 55), their function is often impaired in the TME through interactions with stromal cells, particularly CAFs (56, 57), which play a significant role in modulating DC activity and contributing to immune evasion and tumor progression.

CAFs critically influence DCs within the TME by impairing the antigen-presenting function of DCs and downregulating co-stimulatory molecule expression (such as CD80, CD86, and MHC-II) on their surface. These molecules are crucial for the activation of T cells and the initiation of an effective anti-tumor immune response. This CAF-DC crosstalk involves both the secretion of cytokines, chemokines, growth factors, and extracellular vesicles, along with direct cellular contacts (40). These molecular mechanisms undermine DC functionality, leading to immunosuppression.

**Figure 2:**
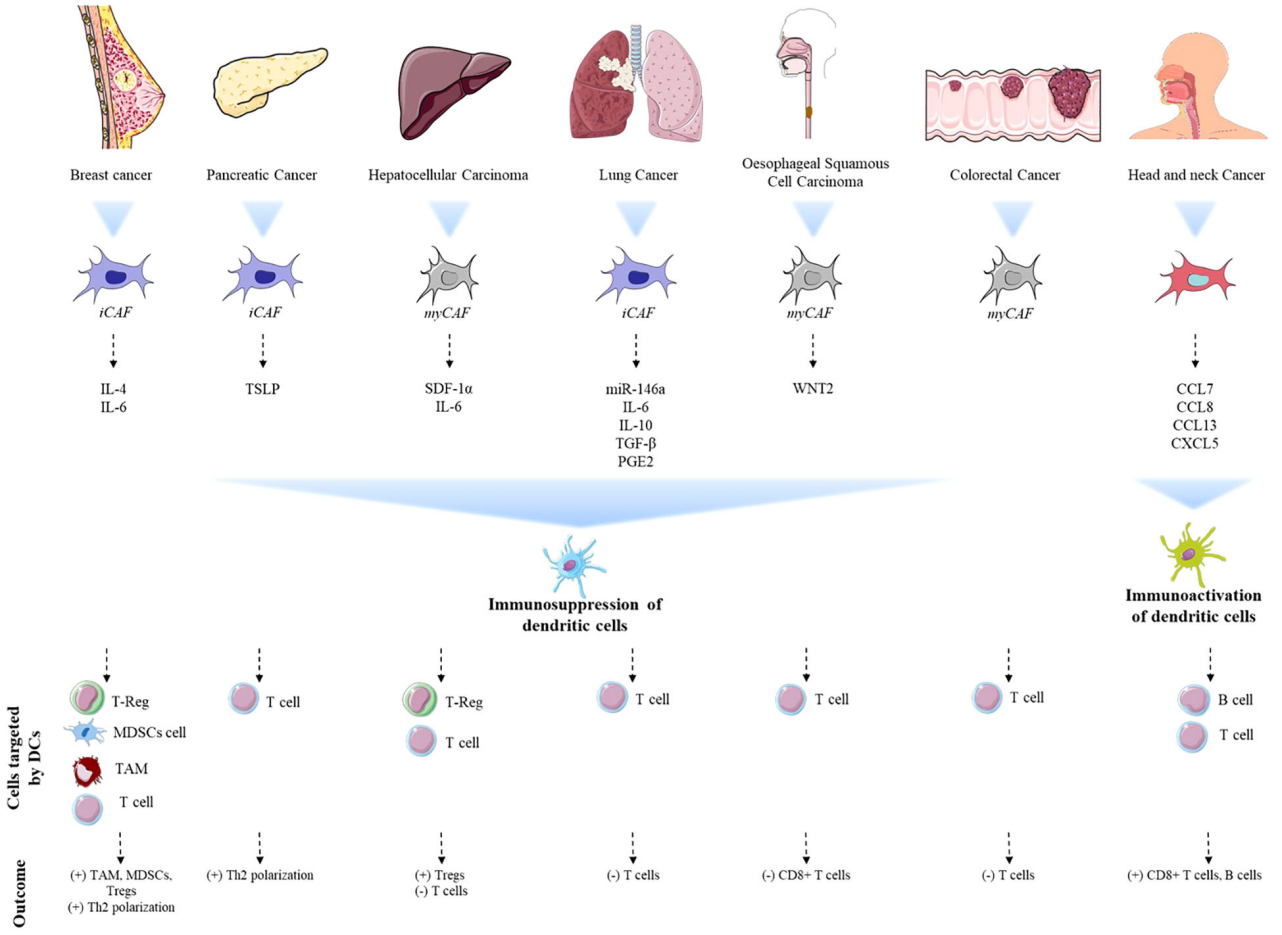
Schematic representation of tumor types and their impact on CAF-DC interactions. Overview of tumor types studied for CAF-DC interactions, highlighting the signaling molecules involved and their impact on DC functionality. The assignment of CAF phenotypes across tumor types, presented in italics, is speculative and based on literature-informed interpretation, not on direct experimental evidence. This figure illustrates how different tumor microenvironments influence the behavior of both CAFs and DCs, emphasizing the complexity of their interactions and the subsequent effects on immune responses. In breast cancer, stromal cells (iCAFs) secrete IL-6 and IL-4, helping tumor growth and immune system modulation by shifting the immune response towards Th2 and attracting pro-tumor cells. Similarly, in pancreatic cancer, iCAFs promote a transition to a Th2 response by secreting TSLP, which makes DCs adopt an immunosuppressive phenotype. MyCAFs from hepatocellular carcinoma secrete SDF-1α and IL-6, leading to immune tolerance in dendritic cells by attracting T-reg cells and reducing T-cell growth. In colorectal and esophageal cancers, myCAFs release WNT2, which prevents dendritic cells from maturing and stops T-cell activation. In lung cancer, iCAFs create a tolerogenic phenotype in DCs by secreting immunosuppressive molecules such as IL-6, TGF-β, PGE2, and IL-10, which block T-cell responses. However, in head and neck cancer, CAFs release chemokines like CCL8 and CXCL5, which attract cDCs, boost immune activation, and help recruit B and T lymphocytes. “+” indicates activation or stimulation; “–” indicates inhibition or suppression.

The modulation of DCs by CAFs seems to be tumor-specific, as distinct CAF-derived factors influence DC maturation, antigen presentation, and immune functions. These effects are shaped by the unique characteristics of the TME, with CAFs secreting factors or activating pathways tailored to the immune landscape of each tumor type. Transcriptomic analysis of fibroblasts has revealed significant differences between fibroblasts from various anatomical sites, suggesting that fibroblasts can transfer tissue-specific information to DCs (58, 59). In the following sections, we will detail the reported CAF-DC crosstalk in various tumor types, with a summary of this information provided in **Table 1**, along with an integrative figure to enhance understanding of these complex interactions (**Figure 2**).

**Table 1:** Overview of CAF-DC interactions across different tumor types.

Tumor type	CAF-DC interaction mechanisms	Analyzed cellular event on dendritic cell	Involved molecules	Origin of DC	Origin of CAF	Findings	Reference
Breast Cancer	Direct	Modulation of immune polarization, affecting recruitment	IL-4 IL-6 IL-2 IL-7	Primary mouse tumor tissue culture	Primary mouse tumor tissue culture	Depletion of CAFs via a DNA vaccine targeting FAP increased DC recruitment and shifted immune polarization from a Th2 to a Th1 response. Enhanced chemotherapy-induced recruitment of DCs and CD8+ T cells, decreased IL-6 and IL-4 expression.	Liao et al. (60)
Pancreatic Cancer	Indirect	Cellular polarization, differentiation, and maturation	TSLP	Monocyte-derived dendritic cells from healthy human blood	Primary human tumor tissue culture	CAFs secrete TSLP upon activation by TNF-α and IL-1β from tumor cells. TSLP activates mDCs to promote Th2 polarization, contributing to tumor progression. Increased Th2 cells are associated with worse prognosis. Blocking TSLP may improve survival	De Monte et al. (61)
Hepatocellular Carcinoma	Direct and indirect	Migration, differentiation, and maturation	SDF-1α IL-6	Monocyte-derived dendritic cells from healthy human blood	Primary human normal and tumor tissue culture	CAFs recruit DCs and induce their differentiation into regulatory DCs, which acquire immunosuppressive properties. Treatment with IL-6 increases STAT3 activation, enhancing IDO production and promoting Treg expansion, leading to immune tolerance	Cheng et al. (62)
Lung Cancer	Indirect	Maturation	miR-146a, IL-6, IL-10, TGF-β	Monocyte-derived dendritic cells from healthy human blood	Primary human normal and tumor tissue culture	CAFs-derived exosomes modulate miRNA and cytokine expression, inducing a regulatory phenotype in DCs	Mirza et al. (63)
	Direct and indirect	Differentiation, maturation, and migration	PGE2 IL-6 TGF-β	Monocyte-derived dendritic cells from healthy human blood	Primary human tumor tissue culture	CAFs modulate the differentiation, maturation, and functions of monocyte-derived DCs, promoting a tolerogenic phenotype	Berzaghi et al. (64)
Oesophageal Squamous Cell Carcinoma	Indirect	Differentiation and maturation	WNT2	Bone marrow derived- dendritic cell from mouse tumor	Primary mouse tumor tissue culture	WNT2 suppresses the differentiation and immunostimulatory activities of dendritic cells	Huang et al. (65)
Colorectal cancers	Direct and indirect	Differentiation and maturation	ND	Monocyte-derived dendritic cells from healthy human blood	Primary human tumor tissue culture	CAFs completely suppress the differentiation of DCs from peripheral blood monocytes, while tumor cells significantly inhibit LPS-induced DC maturation. This indicates that tumor cells and CAFs can modulate different stages of the anti-tumor immune response	Saryglar et al. (66)
	Indirect	Differentiation and maturation	WNT2	Bone marrow derived- dendritic cell from mouse tumor.	Primary mouse tumor tissue culture	WNT2 inhibits the differentiation of DCs affecting T cell activation by disrupting the JAK2/STAT3 signaling pathway, leading to diminished anti-tumor immunity	Huang et al. (65)
Head and neck cancers	Indirect	Migration	Chemokines (CCL7, -8, -13, and CXCL5)	Monocyte-derived dendritic cells from healthy human blood	Primary human tumor tissue culture	Strong association between DC migration and the levels of chemokines released by CAFs	Muijlwijk et al. (67)

Summary of interactions between tumor-associated fibroblasts (CAFs) and dendritic cells (DCs) across various tumor types. It details the mechanisms of interaction (direct, meaning direct contact, or indirect, referring to paracrine signaling), the analyzed cellular events, the involved molecules, and the origins of both DCs and CAFs. The findings from each study are presented along with the corresponding references.

### 5.1 CAF-DC crosstalk in breast cancer

In breast cancer, using the 4T1 metastatic mouse model, CAFs have been shown to modulate immune polarization within the TME, promoting tumor progression and metastasis. Liao et al. demonstrated that *in vivo* depletion of CAFs via a DNA vaccine targeting fibroblast activation protein (FAP) shifted the immune polarization from a Th2-dominant to a Th1-dominant response. This was associated with reduced recruitment of pro-tumor immune cells, including tumor-associated macrophages (TAMs), MDSCs, and Tregs, while enhancing the chemotherapy-induced recruitment of anti-tumor immune cells such as DCs and cytotoxic CD8+ T lymphocytes. The vaccine also increased IL-2 and IL-7 protein expression and enhanced the anti-metastatic effects of doxorubicin, further suppressing IL-6 and IL-4 expression. These findings suggest that CAFs represent viable therapeutic targets in metastatic breast cancer. Consequently, tumor angiogenesis and lymphangiogenesis were reduced, improving anti-tumor immune responses and suppressing spontaneous metastasis of 4T1 breast cancer cells (60).

### 5.2 CAF-DC crosstalk in pancreatic cancer

In pancreatic cancer, CAFs have been found to secrete thymic stromal lymphopoietin (TSLP) following activation by tumor-derived TNF-α and IL-1β. Previous studies have linked TSLP with the induction of Th2 responses through DC activation (68). De Monte et al. showed that TSLP secreted by CAFs activates myeloid dendritic cells (mDCs), endowing them with the ability to promote Th2 polarization (61). Specifically, TSLP-containing supernatants from activated CAFs induced mDCs *in vitro* to upregulate the TSLP receptor (TSLPR), secrete Th2-attracting chemokines, and gain Th2-polarizing capacity. Furthermore, TSLP-activated DCs (CD11c+ TSLPR+) were identified in the tumor stroma and draining lymph nodes, but not in non-draining nodes. *In vivo* experiments showed the presence of Th2-attracting chemokines in the tumor and stroma, and the intratumoral infiltration of Th2 cells was found to correlate with CAF-derived TSLP production and reduced patient survival. These findings demonstrate that a TSLP-driven shift towards Th2 immunity plays an active role in tumor progression. A higher Th2/Th1 cell ratio in the tumor stroma is associated with a poorer prognosis in surgically resected patients. Based on these findings, De Monte et al. proposed a model of complex crosstalk between tumor cells, CAFs, DCs, and Th2 cells that promote tumor growth. In this model, pancreatic tumor cells release TNF-α and IL-1β proinflammatory cytokines, which had been previously reported (69–71), stimulating CAFs to secrete TSLP. This TSLP activates and matures resident DCs loaded with tumor antigens. These activated DCs then migrate to the draining lymph nodes, where they prime Th2 cells specific to the tumor antigen. The primed CD4+ Th2 cells are drawn back to the tumor by Th2 chemokines (TARC and MDC). Recruited GATA-3+ CD4+ Th2 cells contribute to tumor progression. This Th2-driven inflammation is associated with reduced patient survival (61). As a potential solution, blocking TSLP production by CAFs could help improve the prognosis of pancreatic cancer.

### 5.3 CAF-DC crosstalk in hepatocellular cancer

Likewise, in hepatocellular carcinoma tumors, Cheng et al. demonstrated that CAFs modulate DCs differentiating them into regulatory DCs, which acquire characteristics of functional immune tolerance (62). A transwell co-culture model confirmed that CAFs have a strong ability to recruit DCs, and neutralizing SDF-1α significantly inhibits the migration of DCs towards CAFs, while neutralizing IL-6 does not have this effect. Additionally, using an *in vitro* cell-to-cell interaction model, it was observed that DCs adhered to CAFs, forming aggregates. These DCs had fewer and shorter dendrites after treatment with lipopolysaccharide (LPS), along with reduced expression of functional markers. They also showed increased secretion of immunosuppressive cytokines like IL-10, TGF-β, and HGF, while the production of IL-12p70 and TNF-β was decreased. These DCs demonstrated a lower ability to stimulate the proliferation of CD3+ T cells and promoted the expansion of CD4+ CD25+ Foxp3+ regulatory T cells (Tregs) by secreting IDO (62). IDO, produced mainly by immune cells recruited by the tumor, particularly DCs (72) is a key enzyme in tryptophan metabolism involved in immune tolerance and immunosuppression in cancer, as it can induce T cell anergy and Treg expansion (73, 74).

Various studies have shown that regulatory DCs in the tumor region express high levels of IDO, contributing to inhibiting anti-tumor immune responses (75, 76). The transcription of IDO has been linked to the activation of STAT3 (77). In this sense, Cheng et al. also demonstrated that STAT3 activation in DCs, mediated by IL-6 from CAFs, is essential for IDO production. Inhibitory antibodies targeting STAT3 and IL-6 can reverse the regulatory function of CAFs on DCs (62). Furthermore, it was shown that DCs treated with IL-6 exhibited increased STAT3 activation and IDO production, resulting in immunosuppressive effects on T cell responses. In a previous study, IL-6-treated dendritic cells negatively regulated the expression of CD1a, CD83, CD80, CD86, and HLA-DR while increasing CD14 expression (78).

### 5.4 CAF-DC crosstalk in lung cancer

In lung tumors, Berzaghi et al. showed that CAFs paracrinally induced a tolerogenic phenotype in DCs by downregulating key DC markers (CD14, CD1a, CD209) and impairing the differentiation of monocyte-derived DCs *in vitro* (64). The inability of these cells to downregulate CD14 may be related to previous findings showing that IL-6 secretion by CAFs promotes monocyte differentiation into macrophages instead of DCs by inducing M-CSF (79). Additionally, previous studies have shown that high IL-6 expression in stromal cells induces a tolerogenic DC phenotype in a prostate cancer model, characterized by elevated levels of CD14 and PD-L1 (80). The continuous secretion of immunosuppressive signals (IL-6, PGE2, TGF-β) by irradiated lung CAFs may explain why monocyte-to-DC differentiation is unaffected after irradiation (IR) (64, 81, 82). IR has been found to elicit substantial immunological responses that significantly influence disease outcomes in both pre-clinical and clinical settings (83–85). IR induces immunogenic cell death (ICD) and promotes the release of tumor-associated antigens and immune adjuvants, triggering pro-inflammatory reactions and enhancing immune cell recruitment. This process disrupts the equilibrium of tumor immune tolerance (86, 87). However, IR can also activate immunosuppressive pathways that contribute to tumor radioresistance (88). Therefore, treatment outcomes depend on the intricate interplay between pro-immunogenic and anti-immunogenic signals. Recent studies have identified a correlation between CAFs and heightened resistance to radiotherapy in both non-small cell lung cancer (89) and colorectal cancer (90). Furthermore, there is emerging evidence indicating a loss of pro-tumorigenic functions in CAFs following IR exposure (91).

It has also been observed that both paracrine factors from CAFs and cell contact-mediated mechanisms are involved in inducing a tolerogenic phenotype in DCs, characterized by reduced expression of activation markers (CD80, CD86, CD40, and HLA-DR), increased IL-10 release, along with decreased antigen capture, migration capacity, and CD4+ T-cell priming ability (64). This is consistent with previous studies showing that both cell-cell interactions (CAF/DC) and soluble factors secreted by fibroblasts act as strong regulators of DC differentiation and function (92–94).

These findings align with earlier research demonstrating a direct link between tumor-associated fibroblasts and the induction of tolerogenic DCs in hepatocellular carcinoma (62), lung cancers (95), and pancreatic cancer (61). Furthermore, the data suggest that the loss of CAF-mediated effects on DCs following IR is not dependent on the modulation of previously highlighted soluble mediators (TGF-β, IL-6, or PGE2, as well as VEGF, TDO, and TSLP) (61, 95–97).

Berzaghi et al. further explored the impact of ionizing radiation on CAF-mediated regulation of DCs. It has been observed that ionizing radiation, when administered in fractionated medium doses but not in high doses, can modify or reverse CAF-mediated immunoregulatory properties in DCs. This is done by altering CAF paracrine factors that modulate the NF-κB/p65 and STAT3 signaling pathways in DCs. Based on these findings, it is hypothesized that IR may modulate a pro-inflammatory CAF secretome that regulates multiple downstream genes, including cytokines, chemokines, receptors, and transcription factors relevant to dendritic cell functions (64)

In lung adenocarcinomas, CAF-derived exosomes play a critical role in regulating DC maturation by mediating paracrine interactions within the TME. These exosomes modulate miRNA expression and cytokine levels in CAF-conditioned media, contributing to both pro- and anti-tumorigenic responses. Notably, exosomal miRNAs induce post-transcriptional modifications, resulting in epigenetic changes in recipient cells (98, 99). Studies have demonstrated that immature DCs exposed to CAF-conditioned media undergo differentiation into regDCs, marked by reduced expression of maturation markers and an increase in miR-146a levels, underscoring the role of miR-146a as a critical epigenetic regulator in inhibiting DC maturation. This effect was further associated with increased levels of anti-inflammatory cytokines such as IL-6, IL-10, and TGF-β, along with decreased levels of the pro-inflammatory cytokine TNF-α, indicating the suppressive influence of CAF-derived exosomes on DC maturation within the TME (63). Additionally, elevated expression of the regulatory marker CTLA-4 was observed, suggesting its involvement in the generation of regDCs, which suppress T cell responses through the production of IL-10 and IDO (100). Moreover, the potential of curcumin, known for its anti-inflammatory and immunomodulatory effects (101–104), was evaluated in this context. Using ELISA to profile cytokines in CAF-conditioned media treated with curcumin, it was found that curcumin could convert regDCs into mature DCs. These mature DCs were characterized by increased expression of co-stimulatory molecules, reduced CTLA-4 expression, and lower levels of immunosuppressive cytokines and miR-146a. may guide regulatory DCs toward a more mature phenotype, thereby enhancing anti-tumor immune responses. Furthermore, curcumin treatment led to a reduction in IL-6, IL-10, and TGF-β levels in the conditioned media, effectively transforming the TME from an immunosuppressive to an immunomodulatory state. These results align with previous research, demonstrating that curcumin can effectively modulate CAF activity, promoting the secretion of exosomes that create an immunomodulatory TME. This study lays the groundwork for developing synergistic therapeutic strategies combining curcumin with DC-based immunotherapies to overcome cellular resistance in cancer treatment (63).

Despite the immunomodulatory effects of curcumin in preclinical models, its role as a therapeutic agent remains highly debated. While several studies have demonstrated its ability to regulate the TME by reducing immunosuppressive cytokines and promoting DC maturation, significant concerns persist regarding its clinical applicability. However, despite promising laboratory findings, several challenges hinder its translation into clinical practice, including poor bioavailability, rapid metabolism, and variability in systemic absorption. These limitations present major obstacles to its therapeutic use, as high doses are often required to achieve biological effects, raising concerns about potential toxicity and off-target effects. Managing these risks is crucial, and ongoing research aims to find ways to balance therapeutic efficacy with safety, possibly through dose optimization and more targeted delivery strategies. Clinical trials have attempted to address these limitations by testing different curcumin formulations and dosages. Phase I studies have confirmed the presence of curcumin and its metabolites in bodily fluids and tissues, indicating some degree of bioavailability. Moreover, early-stage trials in patients with colorectal, oral, and hepatic cancers have suggested a potential role for curcumin in cancer prevention. However, these initial findings require validation in larger, well-designed studies to determine whether curcumin can meaningfully influence clinical outcomes. Similarly, pilot trials assessing curcumin as an adjunct to conventional therapies have reported benefits such as improved oxidative balance in patients undergoing chemotherapy and radiation, reduced severity of treatment-induced side effects like mucositis and dermatitis, and overall enhancement in quality of life. Yet, the heterogeneity in study designs, patient populations, and dosing regimens makes it difficult to establish standardized recommendations for its use in oncology. One of the major barriers to curcumin’s clinical adoption is its pharmacokinetic profile, which limits its systemic effectiveness. Advances in drug delivery, such as nanoformulations, liposomal preparations, and structural analogs, have been developed to enhance absorption and stability, but their feasibility for widespread clinical application remains uncertain. Additionally, patient-specific factors, including genetic background, gut microbiota composition, and immune status, may influence curcumin’s therapeutic effects, adding another layer of complexity to its use in cancer treatment. Beyond these considerations, curcumin’s broad-spectrum biological activity raises concerns regarding potential unintended effects. While it has demonstrated anti-inflammatory and immunomodulatory properties, these effects could theoretically interfere with certain anticancer treatments, such as immune checkpoint inhibitors and chemotherapy. Its precise role in modulating immune responses within the TME is not yet fully understood, and further research is needed to clarify whether it enhances or counteracts existing therapeutic strategies. Despite these unresolved questions, curcumin continues to be investigated as a potential modulator of immune responses in cancer. Some studies propose that it may help reprogram the TME toward a more immunostimulatory state, which could be beneficial for DC-based immunotherapies. However, until larger, well-controlled trials establish its safety, efficacy, and optimal delivery methods, curcumin cannot yet be considered a standard component of cancer treatment. Future research should focus on refining its pharmacological properties, identifying patient subgroups that may benefit the most, and conducting rigorous clinical evaluations to determine its true therapeutic potential (105).

### 5.5 CAF-DC crosstalk in oesophageal cancer

In oesophageal squamous cell carcinoma (OSCC), WNT2, a secreted glycoprotein that activates the Wnt/β-catenin signaling pathway and promotes tumor progression (106), has emerged as a significant factor in the TME. Notably, WNT2 is primarily expressed in CAFs (106–108). Huang et al. demonstrated that the secretion of WNT2 by CAFs is critical for facilitating tumor immune evasion, as it suppresses DC differentiation, which are essential for activating CD8+ T cells. A negative correlation has been observed between WNT2-expressing CAFs and the activity of CD8+ T cells in primary OSCC tumors, underscoring the role of WNT2 in creating an immunosuppressive microenvironment.

Therapeutic interventions using an anti-WNT2 monoclonal antibody have been shown to increase antigen-presenting DCs within tumors, correlating with enhanced CD8+ T cell responses and tumor suppression. Anti-WNT2 monoclonal antibodies not only restore DC differentiation but also enhance T cell activation, improving the anti-tumor immune response and boosting the efficacy of immune checkpoint inhibitors in tumor models (39). A combination of anti-WNT2 and anti-PD-1 monoclonal antibodies has been found to enhance anti-tumor T cell responses and improve the effectiveness of anti-PD-1 therapy in syngeneic mouse models of OSCC and CRC by increasing active DCs.

At a mechanistic level, WNT2 secreted by CAFs inhibits both DC differentiation and immune-stimulating functions *in vitro*. CAF-derived WNT2 reduces CD11c+ and CD103+ DC differentiation, leading to diminished tumor antigen presentation and CD8+ T cell activation, while not affecting overall CD45+ immune cell infiltration (65). The JAK2/STAT3 pathway, particularly in CD103+ DCs, plays a key role in DC differentiation (109). Studies have demonstrated that the p-JAK2/p-STAT3 (Tyr705) pathway is crucial for DC differentiation, with WNT2 suppressing this process via SOCS3 upregulation in DC precursors. Given these findings, targeting CAF-derived WNT2 could restore DC differentiation and improve T cell responses in tumor models (65). Inhibiting CAF-derived WNT2 could also enhance the efficacy of immune checkpoint inhibitors, such as PD-1/PD-L1 inhibitors, offering new avenues for OSCC immunotherapy. WNT2 also drives tumor growth and invasion by activating Wnt/β-catenin signaling, particularly in OSCC cells. Tumor fibroblast (TF)-derived WNT2 stimulates cancer cell proliferation and invasion through this pathway. *In vitro* studies using CHO-Wnt2 conditioned media have shown that TF-secreted WNT2 promotes tumor cell growth and invasiveness. Targeting WNT2 with monoclonal antibodies presents a potential strategy to disrupt tumor-stroma interactions, reducing tumor growth and metastasis (106).

Beyond OSCC, WNT2 overexpression has been linked to various malignancies, including colorectal (110–114), gastric (115, 116), pancreatic cancer (117), melanoma (118) and non-small cell lung cancers (119). In CRC, WNT2 promotes migration and invasion (107, 111), while in gastric, pancreatic and NSCLC, it accelerates cancer progression (120–122). However, in OSCC, WNT2 expression is primarily restricted to stromal cells, suggesting its role in modulating the TME (106, 123).

While targeting WNT2 is a promising therapeutic strategy, challenges remain across different cancer models. In malignant pleural mesothelioma (MPM), WNT2 expression correlates with poor prognosis and tumor progression. Preclinical studies using human MPM cell lines have shown that WNT2 inhibition via siRNA or monoclonal antibodies induces tumor programmed cell death, even in β-catenin-independent pathways, suggesting a noncanonical role of WNT2 in cell survival. Although anti-WNT2 antibodies alone are less effective than standard chemotherapy like Alimta, their combination enhances tumor suppression (124).

Preclinical studies in breast cancer xenografts demonstrate that WNT2 silencing reduces tumor growth and overcomes chemotherapy (125). In human melanoma models, monoclonal antibodies targeting WNT2 induce apoptosis in WNT2-overexpressing melanoma cells, sparing normal cells. WNT2 siRNA treatment produces similar effects by downregulating β-catenin and survivin, leading to tumor suppression in xenograft models (126).

Despite promising results, translating WNT2-targeted therapies into clinical practice faces significant challenges. Clinical trials remain in early stages, and further research is needed to assess feasibility and safety. Key challenges include potential off-target effects on healthy tissues, efficient tumor delivery, and patient-specific variations in WNT2 expression and immune response. Scaling up production and ensuring consistent therapeutic efficacy also require optimization.

Future research should prioritize early-phase clinical trials to assess safety, dosing, and efficacy of anti-WNT2 monoclonal antibodies in OSCC and other cancers. Combination strategies with immune checkpoint inhibitors or standard chemotherapy warrant further investigation to determine potential synergistic effects. Identifying biomarkers predictive of patient response could help tailor WNT2-targeted therapies, optimizing treatment outcomes. Nevertheless, ongoing preclinical and clinical studies suggest that targeting WNT2 could become a key tool in precision cancer therapy.

### 5.6 CAF-DC crosstalk in colorectal cancer

In colorectal cancer (CRC), WNT2 has also emerged as a pivotal factor in the tumor microenvironment. Similar to OSCC, it is secreted primarily by CAFs and influences tumor dynamics significantly. Huang et al. have shown that WNT2 in CRC acts by inhibiting the differentiation of DCs, thereby impeding T cell activation. Specifically, CAF-derived WNT2 disrupts the JAK2/STAT3 signaling pathway, essential for maintaining DC differentiation, leading to diminished anti-tumor immunity. Targeting WNT2 secreted by CAFs in CRC is proposed as an effective strategy to restore DC differentiation and boost T cell responses in preclinical models, similar to findings in OSCC (65).

Moreover, Saryglar et al. using co-culture experiments demonstrated that colorectal adenocarcinoma cells and stromal cells exert distinct effects on the differentiation and maturation of DCs *in vitro*. CAFs were found to completely suppress the differentiation of DCs from peripheral blood monocytes induced by granulocyte-macrophage colony-stimulating factor (GM-CSF) and IL-4, while having no significant impact on their maturation in the presence of bacterial lipopolysaccharides (LPS). In contrast, tumor cell lines did not impede monocyte differentiation; however, certain lines significantly diminished CD1a expression and inhibited LPS-induced DC maturation. These findings suggested that tumor cells and CAFs may modulate different stages of the anti-tumor immune response (66)

### 5.7 CAF-DC crosstalk in head and neck cancer

In head and neck squamous cell carcinoma (HNSCC), the crosstalk between CAFs and DCs shows a unique pattern compared to other cancers, where CAFs typically drive immunosuppression. In HNSCC, CAFs may instead facilitate immune activation by promoting DC recruitment to the TME. Muijlwijk et al. used an *in vitro* transwell migration assay to show that conventional dendritic cells (cDCs) and B cells, but not CD4+ or CD8+ T cells, were attracted by tumor microenvironment (TME)-conditioned media from different anatomical sites. The cDC migration was site-specific and correlated with distinct proteins in the TME secretome. Further validation confirmed that chemokines like CCL8, CXCL5, CCL13, and CCL7 were responsible for cDC1 and cDC2 migration. Single-cell RNA sequencing showed that CAFs expressed these chemokines, with myCAFs having the highest CXCL5 levels. Removing fibroblasts from the TME reduced DC migration, showing that, even though CAFs are usually seen as immunosuppressive, they actually help attract cDCs to the TME in HNSCC. This recruitment could be important for better antitumor immunity and treatment response. Additionally, patients who responded well to immune checkpoint inhibitors (ICIs) showed higher RNA expression of these chemokines, cDCs, myCAFs, and iCAFs compared to non-responders. This suggests that certain CAF subtypes may play a key role in attracting cDCs and improving responses to immunotherapy (67)

As discussed throughout this review, the interaction between CAFs and DCs plays a crucial role in the TME across various cancers. Understanding the distinct characteristics, functional roles, and cytokine profiles of specific CAF subtypes offer valuable insights into their contributions to tumor progression. It is reasonable to suggest that CAFs adopt unique phenotypes depending on the tumor type, thereby modulating DC activity in distinct ways. From the analysis, it becomes clear that the CAF phenotypes present in each tumor type dictates their immunomodulatory roles. In tumors where iCAFs are dominant, immune suppression is mediated primarily through cytokine signaling, influencing DC maturation and functionality. These CAFs are characterized by secretion profiles of inflammatory cytokines that drive immune evasion. The degree of CAF heterogeneity complicates the assignment of specific subtype associations, especially when comprehensive profiling data is lacking. In the reported breast cancer study using an orthotopic tumor model, the authors demonstrated that IL-6 and IL-4 secretion by these stromal cells facilitates tumor progression and immune modulation (60). Over the past decade, several heterogeneous subsets of CAFs have been identified, particularly from human breast tumors. Four distinct subsets have been characterized, each defined by the expression of a specific range of markers (51). Nevertheless, the aforementioned study did not provide an extensive characterization of the CAF subtypes present in the orthotopic breast cancer model. However, one of the subtypes described by Costa et al. aligns closely with the reported immune signature and the activated phenotype associated with the expression of the protein FAP (51). This strongly suggests that the myCAF population could play a significant role in this solid tumor.

Similarly, in pancreatic cancer, CAFs facilitate a shift toward a Th2 immune response through the secretion of TSLP in response to TNF-α and IL-1β, which polarizes DCs toward an immunosuppressive phenotype (61). Given that these CAFs promote inflammation and an immunosuppressive response, this aligns with the characteristics of iCAFs, which are known for secreting pro-inflammatory cytokines such as IL-6, further emphasizing their role in modulating the immune microenvironment through cytokine-mediated pathways that induce tolerogenic DCs. A controversial situation was observed in primary cultures derived from fresh early-stage prostate carcinoma (PCa). This study elucidated the role of the PCa-associated stroma, characterized by a myofibroblastic phenotype, marked by a strong presence of α-SMA, in contributing to an immunosuppressive microenvironment. The defined role of this CAFs subtype is linked to the secretion of cytokines, such as IL-6, which play a pivotal role in influencing the differentiation of myeloid cells into conventional DCs exhibiting an immunosuppressive phenotype. Moreover, in prostate cancer, the secretion of IL-6 by CAFs indicates a predominance of iCAFs (80).

The myCAFs subtype engages in direct cell-cell interactions, physically reshaping TME while further inhibiting immune activation. In a study utilizing CAFs derived from hepatocellular carcinoma, the physical contact between CAFs and DCs resulted in the induction of functional immune tolerance in the DCs (62). This stromal population, previously classified as myofibroblasts (127), was shown to recruit DCs through an SDF-1α-dependent mechanism, subsequently educating them to adopt a tolerogenic phenotype via IL-6-mediated STAT3 activation. These findings suggest that the interaction between myCAFs and immune cells is not solely dependent on physical interactions (62). This direct modulation type is characteristic of myCAFs, which often exert their influence via physical interactions rather than solely through soluble factors. In colorectal and oesophageal cancers, CAFs secrete WNT2, a factor that inhibits DC differentiation and T cell activation, thereby facilitating immune evasion (65). This observation, however, does not fully align with the classification of one CAF subtypes, where low levels of WNT2 are associated with the iCAF phenotype, while high levels are linked to the myCAF phenotype (128).

In lung cancer, CAFs derived from freshly resected tissue have been shown to secrete immunosuppressive cytokines such as IL-6, TGF-β, and IL-10, which contribute to the immune suppression observed in the tumor microenvironment. This suppression is mediated through both paracrine signaling and direct cell-cell contact mechanisms (63, 64). The validation of the specific CAF subpopulation involved in this process remains crucial. In this context, the molecular signatures of inflammatory iCAFs are particularly important, as they play a central role in inducing tolerogenic DC phenotypes. The capacity of CAFs to mediate immune suppression strongly correlates with the iCAF phenotype (41), reinforcing the idea that iCAFs are primary contributors to the generation of immune tolerance within lung cancer microenvironment.However, in head and neck cancer, a notable exception arises. Here, CAFs secrete chemokines like CCL8 and CXCL5, attracting cDCs into the tumor microenvironment (67). This recruitment potentially boosts antitumor immunity and improves responses to immune checkpoint inhibitors (ICIs), suggesting that, contrary to their typical immunosuppressive roles in other cancers, CAFs in this setting may facilitate immune activation, highlighting a distinct functional shift in their role within this specific tumor microenvironment. This underscores the potential of modulating CAF activity to promote immune-stimulatory roles, which is a crucial objective in the advancement of future cancer therapies.

In conclusion, the interaction between CAFs and DCs plays a critical role in shaping the immune landscape of the tumor microenvironment. The gene profile of distinct CAF subtypes contributes to their functional diversity, exerting immunomodulatory effects that vary depending on the cancer type. The inherent heterogeneity and plasticity of fibroblasts enable them to dynamically transition between immunosuppressive and immune-stimulatory roles, depending on the tumor context. While these correlations are speculative, they are grounded in current biological and molecular knowledge and aim to provide a conceptual basis for future studies. Experimental validation will be essential to confirm or refute these associations and to clarify their therapeutic relevance. As highlighted in this review, targeting specific CAF subpopulations in certain cancers presents promising opportunities to enhance the efficacy of immunotherapies and to develop more effective cancer treatments.

## 6 Clinical translation of DC- and CAFs based immunotherapies

DC-based immunotherapies have been extensively studied for their ability to elicit robust anti-tumor immune responses. The most widely used therapeutic strategy involves *ex vivo* differentiation of autologous monocytes into DCs (mo-DCs), followed by antigen loading with tumor-associated (TAAs) or tumor-specific antigens (TSAs), maturation, and reinfusion into the patient (129, 130). This process aims to stimulate and enhance the activity of effector immune cells, thereby promoting the elimination of tumors. Naturally occurring DCs are also employed in these therapies, albeit to a lesser extent than mo-DCs (131). Preliminary results indicate that these regimens are generally safe and well-tolerated, with the most common adverse events being mild to moderate (grade 1 to 2) symptoms. These often include flu-like effects that resolve quickly, such as fever, fatigue, and chills, as well as localized reactions at the injection site (131).

Numerous clinical trials have evaluated DC vaccines across several tumor types, including melanoma, glioblastoma, prostate, lung, and ovarian cancers (132–134). These studies primarily employed autologous monocyte-derived DCs (moDCs), with antigen-loading strategies based on peptides (135), tumor lysates (136) or RNA (137). Sipuleucel-T (Provenge^®^) remains the only FDA-approved DC-based immunotherapy to date, for castration-resistant prostate cancer (138), although its clinical impact has been modest.

To enhance therapeutic outcomes, recent strategies explore combination therapies involving immune checkpoint inhibitors (ICIs), adoptive T cell transfer (ACT), and neoantigen-based targeting (139, 140). Additionally, the use of naturally occurring DC subsets, such as cDC1, cDC2, and pDCs, is gaining interest due to their superior antigen-presenting capabilities compared to moDCs (26, 141). Clinical trials are also investigating *in vivo* DC activation and genetically engineered DCs.

DC vaccines are currently being evaluated in tumors with poor responsiveness to other immunotherapies, such as glioblastoma and immune-cold cancers (142, 143). While these vaccines generally exhibit favorable safety profiles (144), therapeutic efficacy varies widely due to patient heterogeneity, differences in immune competence, and antigen presentation dynamics (145).

In parallel, CAFs are being explored as targets for immunotherapy. Three main therapeutic strategies are under investigation: CAF depletion, functional inhibition, and phenotype reprogramming. Among the CAF targets, fibroblast activation protein (FAP) is the most extensively studied. Agents such as talabostat and sibrotuzumab showed early promise in preclinical models (146), but failed to yield significant clinical benefits in trials as monotherapy (147). More recently, radiolabeled FAP inhibitors (FAPI) have demonstrated selective tumor targeting and encouraging early clinical outcomes (148, 149).

Beyond FAP, other CAF-associated proteins like PDGFRα/β and FSP-1 are under investigation. Imatinib (a tyrosine kinase inhibitor targeting PDGFR) has shown potential in combination regimens, while niclosamide (an FSP-1 inhibitor) is currently being tested in clinical trials (149, 150). Reprogramming strategies, such as the use of vitamin A or D to modulate TGF-β signaling, offer alternative avenues to inhibit CAF-mediated immunosuppression without inducing adverse pro-tumorigenic effects (151).

Multitargeted agents such as Nintedanib, which inhibits VEGFR, FGFR, and PDGFR, are also being evaluated for their ability to modulate CAF functions and improve responses to chemotherapy and immunotherapy in cancers like NSCLC (152). Although CAF-targeted therapies have not yet achieved consistent clinical success, their integration into combination regimens and ongoing refinement of preclinical models offer a promising path forward (149).

## 7 Addressing key research gaps

A substantial body of scientific evidence positions CAFs as pivotal players in tumor progression. An insightful review titled “Accessories to the Crime” illustrates how CAFs are not merely passive components of the TME; rather, they actively collaborate with tumors in adopting each hallmark characteristic that defines cancer as a disease of high morbidity and mortality (5).

The advent of immunotherapy has revolutionized cancer treatment, leading to significant improvements in survival rates for many patients (153, 154). This paradigm shift is particularly evident in tumors classified as immunologically “hot” which are characterized by a robust immune cell infiltration and an active immune response against the tumor. Immunotherapy leverages the body’s immune system to identify and attack cancer cells, with immune checkpoint inhibitors (ICIs) being one of the most promising and widely studied classes of these therapies (155). ICIs function by targeting specific regulatory pathways that cancer cells exploit to evade immune detection. By inhibiting these pathways, ICIs can reinvigorate exhausted T-cells, enhancing their ability to recognize and destroy cancer cells. Clinical trials have demonstrated the efficacy of ICIs in various malignancies, including melanoma, non-small cell lung cancer, and renal cell carcinoma, resulting in durable responses and prolonged survival for a subset of patients (156). However, the effectiveness of ICIs is not universal; they tend to yield limited results in certain tumor types, particularly those where CAFs are predominant, such as PDAC (157). In these tumors, the immunosuppressive environment created by CAFs presents a significant barrier to the success of ICI therapies.

CAFs not only physically obstruct immune cell infiltration but also secrete a variety of cytokines and growth factors that further promote immune suppression (40). Consequently, the question arises: while existing therapeutic strategies targeting CAFs are beginning to emerge (158), how can we enhance and refine these approaches to more effectively overcome the challenges posed by CAFs in cancer therapy?

In this review, we focus on the detailed analysis of how CAFs can modulate the phenotype and functionality of DCs. These cells play a crucial role in both immunotherapy and tumor immunology, acting as intermediaries between innate and adaptive immune responses. DCs are key players in orchestrating immediate and long-lasting protective immune effects, whether endogenous or induced, against tumors (22, 24). The critical involvement of CAFs in inducing a tolerogenic program within DC populations raises important questions about their potential implications in the therapeutic failures observed with DCs-based vaccination strategies.

At its core, there is an expectation of significant success from dendritic cell (DC)-based therapeutic approaches, given the essential role of DCs in the immune response (131, 159). DCs are frequently utilized in clinical trials due to their status as premier antigen-presenting cells, making them an ideal vehicle for antigen administration. However, despite these encouraging results and the absence of high-grade adverse events, the clinical application of therapeutic vaccines based on DCs differentiated from autologous monocytes of cancer patients, as well as those derived from naturally occurring DCs, has not demonstrated a significant improvement in survival outcomes across various tumor types (160). This failure is strongly associated with the dysfunction, immunosuppression, and tolerogenic phenotype exhibited by their precursors, as well as the DCs that differentiate from them (161–166). This is in stark contrast to DCs derived from healthy donors, which generally exhibit more effective immunogenic profiles. Naturally occurring DCs, while also employed in therapeutic settings, face similar challenges related to their functionality in the tumor microenvironment (131). Thus, we are compelled to ask: can we reverse the tolerogenic state of both mo-DCs and naturally occurring DCs derived from cancer patients to apply them safely and effectively in a therapeutic vaccine?

Numerous signals inherent to the TME have been identified as capable of inducing tolerogenicity (167, 168). Our hypothesis posits that these signals are enriched in the presence of tumors with high CAF levels. As current therapeutic strategies primarily focus on tumor removal, the residual presence of CAFs may facilitate the persistence of immunosuppression following tumor excision.

Moreover, it is crucial to note that immunosuppression in cancer patients has been reported to be systemic (169). Therefore, when reconsidering the development of DC- vaccines, understanding the signals and events involved in this process is fundamental for developing therapeutic strategies that counteract these unwanted effects. The significance of CAFs in these processes cannot be overstated, particularly given the limited options currently available to target them effectively. may further aid in this endeavor. A preliminary report supports this idea, demonstrating that the use of anti-fibrotic (tranilast) agents as an anti-CAF strategy (170) shows promise in enhancing the efficacy of DC vaccines by targeting the immune TME. Notably, immunization of mice with DCs transfected with fibroblast activation protein (FAP) mRNA led to significant antitumor responses, highlighting FAP as a potential tumor rejection antigen in a variety of cancers (171).

On the other hand, restoring the immunogenicity of DCs emerges as a crucial goal for achieving therapeutic success. Our research team is fervently dedicated to investigating this critical issue, with a particular focus on the induction of immunogenic tumor cell death (ICD) as a promising mechanism to enhance both tumor adjuvanticity and antigenicity (172–176). By leveraging this approach, we aim to generate a robust immune response that not only activates effector immune cells but also effectively counteracts the immunosuppressive effects induced by TME, in particular by CAFs (177–179). The concept of ICD in cancer involves not only the elimination of tumor cells but also the exposure and release of tumor antigens, which are crucial for enhancing antigenicity, which represents the ability of these antigens to provoke an immune response. This process facilitates the recognition of tumor antigens by the immune system, thereby promoting a more vigorous and effective anti-tumor response. Furthermore, the adjuvanticity of the induced tumor cell death is essential, as it amplifies the immune response by creating a more favorable environment for immune cell activation and proliferation (86). In terms of DC-vaccination protocols, this approach could represent a better source of antigens/adjuvants for stimulation, thereby counteracting the patient’s inherent immunosuppression (172). By enhancing both antigenicity and adjuvanticity, this strategy holds the potential to mitigate the immunosuppression exerted by the tumor microenvironment, ultimately leading to improved therapeutic outcomes. In this context, Berzaghi’s work highlights how IR, which was previously linked to ICD (172), partially reversed the immunosuppressive effects exerted by CAFs on DCs. Their study shows that while CAFs typically induce a tolerogenic phenotype in DCs, certain radiation protocols disrupt this effect, improving DC functionality and reducing the expression of key immunosuppressive markers (64). This finding suggests that ICD-inducing therapies like radiation could effectively counteract the suppressive influence of CAFs, enhancing immune activation and leading to more effective cancer immunotherapies.

Despite the potential of this strategy, there remains much to uncover in this field. We need to deepen our understanding of the intricate interactions between tumor cells, immune cells, and CAFs. Additionally, the mechanisms by which optimal ICD can be induced and maintained within the tumor microenvironment are still not fully elucidated. Our ongoing research aims to address these gaps, exploring various modalities and treatments that could synergistically enhance the immune response while diminishing the immunosuppressive barriers posed by CAFs. Ultimately, we seek to identify innovative therapeutic strategies that can be translated into clinical applications, paving the way for more effective cancer treatments.

## 8 Conclusion

The dynamic interactions between CAFs and DCs play a pivotal role in shaping the TME and influencing immune responses. CAFs exert a profound immunosuppressive effect on DCs, impairing their ability to initiate effective antitumor immunity by disrupting antigen presentation and promoting tolerogenic phenotypes.

A comprehensive characterization of CAF subtypes in tumor-specific contexts will provide a more nuanced understanding of how these interactions influence immune modulation, paving the way for the development of cluster-specific therapeutic strategies. Additionally, resolving the mechanisms that determine whether CAFs promote immune tolerance or immune activation will help identify optimal therapeutic windows for targeting CAFs in combination with immunotherapies.

To move the field forward, a clear research agenda is needed, one that focuses on: (i) defining the tumor-specific functional impact of distinct CAF subtypes on DC phenotype and function; (ii) identifying the signaling pathways that mediate CAF-DC crosstalk; (iii) understanding how these interactions influence the efficacy of DC-based and combinatorial immunotherapies; and (iv) prioritizing therapeutic targets capable of reprogramming the CAF-DC axis toward immunostimulatory outcomes. Advancing this agenda will contribute to the development of more precise and effective immunotherapeutic strategies, ultimately improving patient outcomes.
